# ImmunoSPdb: an archive of immunosuppressive peptides

**DOI:** 10.1093/database/baz012

**Published:** 2019-02-08

**Authors:** Salman Sadullah Usmani, Piyush Agrawal, Manika Sehgal, Pradeep Kumar Patel, Gajendra P S Raghava

**Affiliations:** 1 Department of Computational Biology, Indraprastha Institute of Information Technology, New Delhi, India; 2 Bioinformatics Centre, CSIR-Institute of Microbial Technology, Chandigarh, India

## Abstract

Immunosuppression proved as a captivating therapy in several autoimmune disorders, asthma as well as in organ transplantation. Immunosuppressive peptides are specific for reducing efficacy of immune system with wide range of therapeutic implementations. `ImmunoSPdb’ is a comprehensive, manually curated database of around 500 experimentally verified immunosuppressive peptides compiled from 79 research article and 32 patents. The current version comprises of 553 entries providing extensive information including peptide name, sequence, chirality, chemical modification, origin, nature of peptide, its target as well as mechanism of action, amino acid frequency and composition, etc. Data analysis revealed that most of the immunosuppressive peptides are linear (91%), are shorter in length i.e. up to 20 amino acids (62%) and have L form of amino acids (81%). About 30% peptide are either chemically modified or have end terminal modification. Most of the peptides either are derived from proteins (41%) or naturally (27%) exist. Blockage of potassium ion channel (24%) is one a major target for immunosuppressive peptides. In addition, we have annotated tertiary structure by using PEPstrMOD and I-TASSER. Many user-friendly, web-based tools have been integrated to facilitate searching, browsing and analyzing the data. We have developed a user-friendly responsive website to assist a wide range of users.

## Introduction

Immune regulation is a complex and essential procedure to resolve infection, inflammation and endorsement of immunological memory. Immune system is continuously under surveillance to control and maintain the homeostasis. It detects and eliminates tumor cell, pathogens as well as foreign entities, and also avoids self-recognition (1, 2). But sometimes, failure to immune regulation results into over reactiveness of immunological tools, which leads to allergy as well as autoimmunity. According to American Autoimmune Related Disease Association, 50 million Americans suffer from autoimmune diseases and it is one of the top 10 foremost death causes in women, in up to 64 years of age groups. Approximately 80–100 different autoimmune diseases such as rheumatoid arthritis, Hashimoto thyroiditis, Graves' disease, type 1 diabetes mellitus etc. have been identified. Researchers also suspect at least 40 additional chronic and life-threatening diseases of having an autoimmune basis (https://www.aarda.org/news-information/statistics/). Worldwide, the mean ± SD were 19.1 ± 43.1 and 12.5 ± 7.9 for the net percentage increased per year incidence and prevalence of autoimmune diseases, respectively (3).

Beside autoimmune disease, there are other life-threatening illnesses in which organ transplantation must be enforced to save the life. According to “U.S. Government Information on Organ Donation and Transplantation”, 20 people die each day waiting for a transplant, irrespective of 80 people receive organ transplant (https://www.organdonor.gov/statistics-stories/statistics.html). Organ transplantation is the best therapy in irreparable organ failure and has gradually amended in the past two decades, providing better results in children as compared to adults, as matured host immune system tends to reject the foreign organ (4). In these scenarios immunosuppression i.e. targeted reduction in the efficacy of immune system is necessary to execute organ transplantation. Many immunosuppressive agents have already been used for the treatment of autoimmune disease, allergy and asthma and after organ transplantation (5, 6). Some of these pharmaceuticals interfere with gene expression, inhibit nucleotide-base synthesis to block cell cycle or target specific kinase. These molecular targets have disadvantages of affecting the whole immune system, resulting in severe therapeutic as well as non-therapeutic toxicity such as cardiotoxicity, nephrotoxicity and post-transplant diabetes mellitus (7, 8).

In the past two decades, there is a paradigm shift toward peptide-based therapy as compared to small molecule-based drug development due to less cytotoxicity, high efficacy as well as high specificity revealed by therapeutic peptides, by the virtue of their inherent molecular targeted action (9, 10). High efficacy as well as easy and economical synthesis of peptide also draws attention of scientific community as well as biotech industry (11, 12). Peptides have been studied for various therapeutic property such as antimicrobial, antifungal, antibacterial, antimycobacterial, cell penetrating, cancer-biomarker etc. (13–17). Similarly, a large number of peptides have been studied for their ability to suppress the immune system and emerged as promising immunosuppressive therapeutic entity due to their oral administration property and remarkable stability as exhibited by antamides, cyclosporine A (CsA) etc.

Most of the immunosuppressive peptides are shorter in length (<40 amino acid), are linear and have natural origin such as fungus, venom toxin as well as plants and derived from an existing protein. CsA is a cyclic peptide originated from fungus *Trichoderma polysporum,* is discovered in 1976 and has been the principal immunosuppressive agent used in the medicine (18). Charybdotoxin, magatoxin, kaliotoxin etc. are derived from the venom of scorpion and are potential potassium channel blocker (19). Beside this, several plant orbitides such as cyclolinopeptides and cyclotides as katala B1 have been known for their immunosuppressive activities (20, 21). These peptides have diverse mechanism to suppress the immune system, but generally they are targeted against a specific signaling molecule and thus having fewer side effects (19). Despite of its huge therapeutic importance, there is no repository of immunosuppressive peptides till date, and thus, it is very difficult to access the scattered information from the literature. In order to assist the scientific community, we have endeavored to compile all the scattered information and developed an archive of immunosuppressive peptides—`ImmunoSPdb’.

## Material and methods

### Data acquisition

Immunosuppressive peptides were collected from research articles and patents. Combination of keywords like ‘immunosuppressive peptide’, ‘peptide based immunosuppression’, and ‘immunosuppressive agent’ in PubMed advanced search criteria while selecting `Title/Abstract’ resulted into approximately 3400 articles. Similarly, patents were searched from United States Patents and Trademark Office (USPTO) using keywords ‘immunosuppressive peptide’ and ‘peptide based immunosuppression’ which resulted into 58 patents. All articles were manually screened for relevant information and final data were compiled from 79 research article and 32 patents. In total, ImmunoSPdb includes 553 entries of 498 unique immunosuppressive peptides.

### Database architecture and web interface

**Figure 1:**
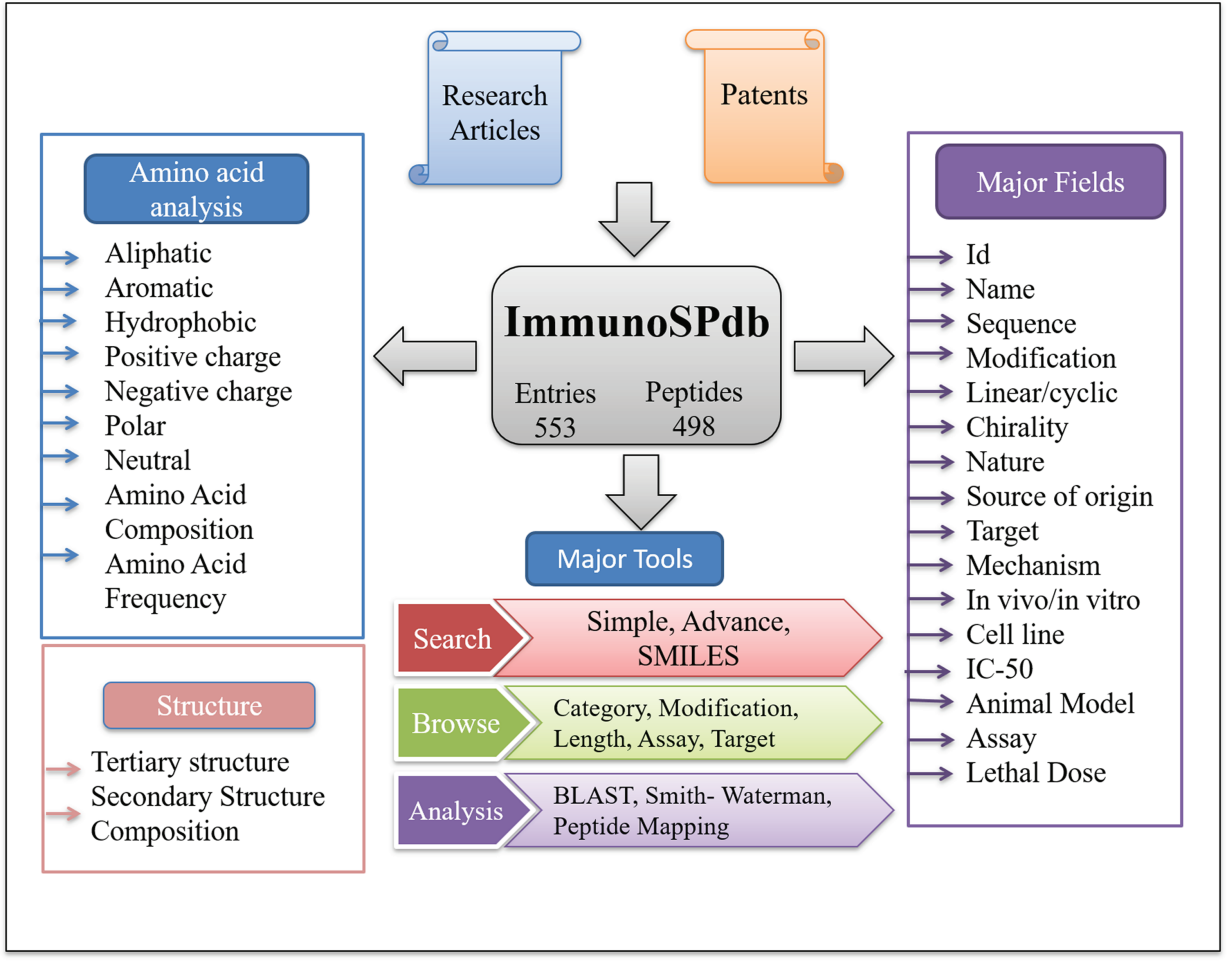
Architecture of ImmunoSPdb.

ImmunoSPdb has been built on Linux-Apache-MySQL-PHP (LAMP)-based standard platform. Ubuntu (version 14.04.5) as the operating system, MySQL (version 5.5.55) for managing the data and Apache (version 2.4.7) as the HTTP server were used. HTML5, PHP5, CSS3 and JAVA scripts have been used for developing responsive front ends, which are compatible for mobile, tablets and desktop. PHP and PERL coding have been explored for the entire database interface, common gateway and networks. The architecture of ImmunoSPdb database is given in Figure 1.

### Database organization

#### Primary information

General information about immunosuppressive peptides can be categorized as primary data. It contains manually extracted information from research article as well as patents. Information has been provided in a tabular form under different headers or fields. Main fields are designated as follows: (i) Name: represents the peptide name used in literature; (ii) Sequence: provides amino acid sequence of immunosuppressive peptide; (iii) Nature: represents the nature (e.g. natural or synthetic or protein derived) of peptide; (iv) Source: represents the sources of origin of peptide; (v) Target: represents target on which peptide act to suppress the efficacy of immune system; (vi) Mechanism of Action: describes the mechanism by which immunosuppressive peptide perform its suppressive activity; (vii) Cell line: represents the cell line used to validate immunosuppressive peptide; (viii) In vivo Model: represents animal model used to check the immunosuppressive activity of peptide; (ix) Assay Type: represents the experimental assays performed to validate immunosuppressive peptide; and (x) Modification: represents the modification, either end modification at N (e.g. Acetylation) or C terminal (e.g. Amidation) or chemical modification (e.g. Disulfide linkage, Modified amino acid etc.)

#### Secondary information

It has been shown that activity of a peptide also depends on its structure (22), so understanding of its structure will be a major advantage to elicit its function. Since most of the peptide doesn’t have known crystalize structure, we therefore have predicted its tertiary structure by using software PEPstrMOD and I-TASSER (23, 24). If the peptide length was 25 or less, it has been predicted by PEPstrMOD, and in case of peptides having length more than 25 amino acids, I-TASSER was used to predict its structure.

In order to better understand the nature of peptide, its properties (as aliphatic, aromatic, polar, hydrophobic, neutral amino acid etc.) were also calculated by using in-house PERL scripts. Beside this, Simplified Molecular Input Line Entry System (SMILES) of each peptide has been generated using Open Babel software (25). The information is very useful to understand which type of residues is preferred in immunosuppressive peptides. All the above information is stored in tabular form as secondary data for easy searching, browsing and analysis of peptides based on their physiochemical properties.

### Implementation of tools

#### Data retrieval or search tools

This module of ImmunoSPdb has been designed to ease effortless searching of the data using simple and advanced search options. The user can give the query against any filed of the database such as name, sequence, IC-50, assay, end terminal and chemical modifications, mechanism of action, cell line, *in vivo* model etc. The simple search module allows the output customized according to the search query. Whereas the user can give multiple queries simultaneously with Boolean expressions (e.g. AND, OR and NOT) in the advanced search module. In addition, a SMILES search facility has been also implemented for ease of the user.

#### Browsing

A user-friendly browsing interface has been developed to ease effortless retrieval of the information. Physicochemical properties such as the hydrophobicity, aliphaticity, aromaticity, negative or positive charge and polar or neutral charge of each immunosuppressive peptide have been computed, which can be easily browsed by the chosen physicochemical properties. Beside this, secondary structure composition of peptides has also been computed and can be used for browsing. Most notably the users can browse on major significant fields: (i) category of peptides (e.g. peptide type, nature and chirality), (ii) modification of peptides (e.g. N- or C-terminal modification and chemical modification), (iii) peptide length, (iv) assay type, and (v) target.

#### Sequence alignment

We have integrated Basic Local Alignment Search Tool (BLAST) and Smith–Waterman algorithm to facilitate sequence similarity-based search (26, 27). In the BLAST module, user have to submit FASTA format of peptide sequence with default or chosen parameters and server automatically performs the BLAST search against the primary structures of all the peptides stored in the database. Similarly, Smith–Waterman algorithm also performs similarity-based search against immunosuppressive peptide sequences. Beside this, we have also integrated two sequence mapping tools, sub-search and super-search, based on identical residues, which allows mapping of peptide sequence against all immunosuppressive peptides as well as identification of identical segments in a query peptide.

## Results

**Figure 2:**
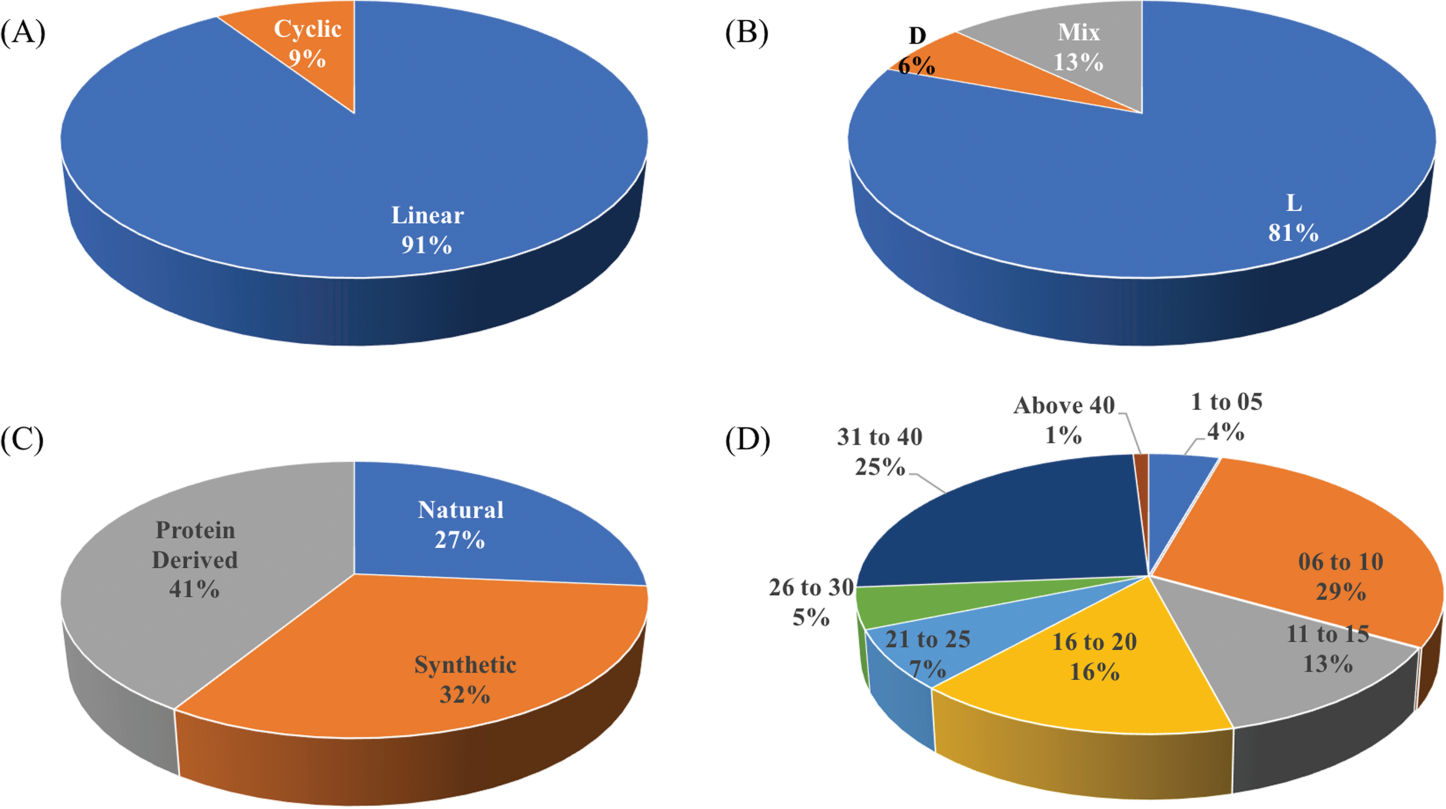
Schematic representation of distribution of peptide entries in ImmunoSPdb based on (A) linear/cyclic conformation, (B) chirality, (C) nature and (D) length of immunosuppressive peptides.

ImmunoSPdb is a unique compilation of 553 entries, containing 498 unique experimentally verified immunosuppressive peptides. The important feature of ImmunoSPdb is that it contains 145 modified unique peptides. Figure 2 shows the distribution of peptide entries that includes approximately 500 linear, 52 cyclic; 443 consist of amino acid having L chirality while 35 have D and 72 having mix chirality of amino acids. ImmunoSPdb contains peptides having natural (146), protein derived (228) or synthetic origin (179). Most of the peptides are short in length, i.e. 174 consist of fewer than or equal to 10 amino acids, 150 having 11 to 20 amino acids, 62 consist of 21 to 30 amino acids and 138 peptides having 30 to 43 amino acids. Interestingly, two peptides that were derived from protein of human immunodeficiency virus were very large, i.e. 146 amino acids and glycodelin-A consist of 180 amino acids. These analyses provide a good insight about the origin of immunosuppressive peptides, as most of them are of naturally occurring and derived from some natural existing proteins. Eight peptides are of natural origin and obtained from the venom of scorpions, such as Vm24 from Mexican *Vaejovis mexicanus smithi*, charybdotoxin from *Leiurusquin questriatus var. hebraeus*, iberiotoxin from *Buthus tamulus*, kaliotoxin from *Androctonus mauretanicus*, margatoxin (or magatoxin) from *Centruroides margaritatus* and OSK-1 from Central Asian *Orthochirus scrobiculosus* etc. CsA from *Trichoderma polysporum*, colutellin A from *Colletotrichum dematium*, antamides from *Tolypocladium inflatum* etc. are originated from fungus. Beside this, several peptides are of plant origin such as katala B1, curcacycline B, cyclonurinin, cyclolinopeptide A and B etc. Microcolin A and B are isolated form blue–green algae whereas cycloamanide A/B/C/D are isolated from mushroom *Amanita phalloides*.

**Figure 3:**
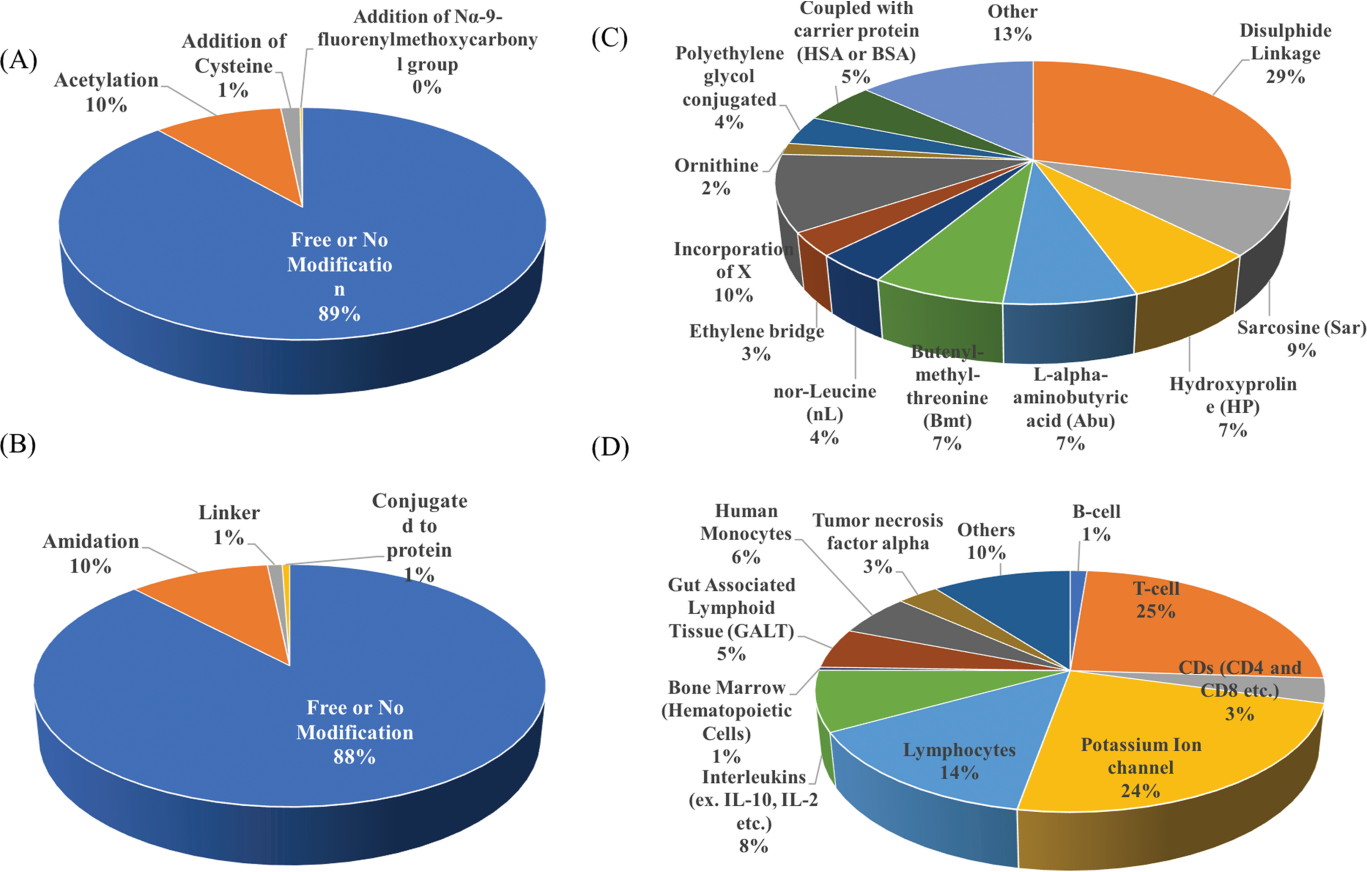
Distribution of peptide entries based on (A) N-terminal, (B) C-terminal, (C) chemical modification and (D) target of action of immunosuppressive peptides.

**Figure 4:**
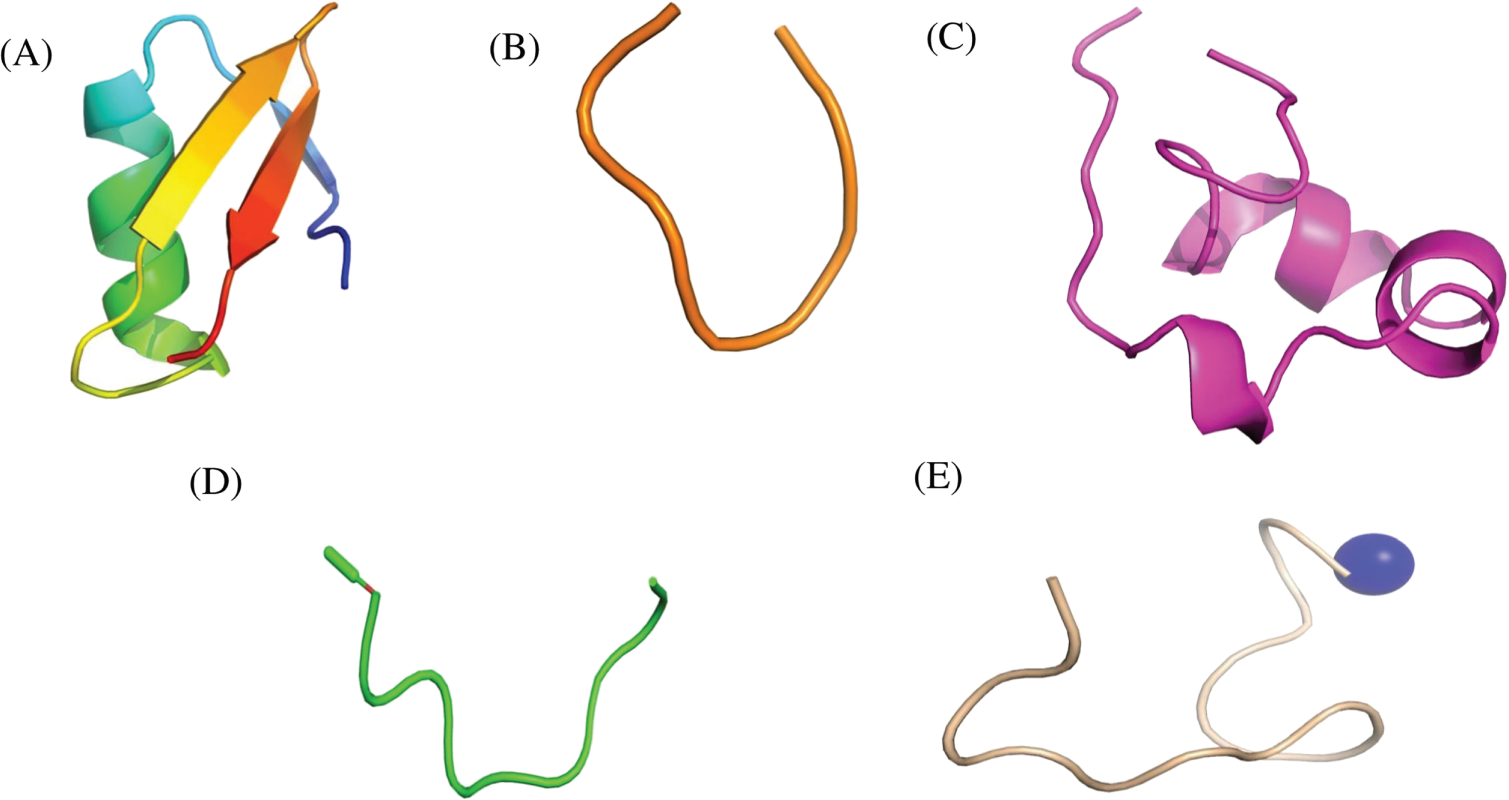
Representative structures of various immunosuppressive peptides: (A) margatoxin having helix and sheets, (B) cycloamanide B having cyclic structure, (C) peptide named as SEQ ID 30 exhibit helical, (D) Ac-1-9 having N-terminal acetylation and (E) H17 having C-terminal amidation stored in ImmunoSPdb.

Researchers have tried to implement end-terminal modification to overcome drawback of a peptide; mostly acetylation at N-terminal end (Figure 3A) and amidation at C-terminal end (Figure 3B). Some naturally occurring peptides have modified amino acids, for example CsA which was first isolated from fungus *Trichoderma polysporum* have certain modified amino acids and their derivatives like butenyl-methyl-threonine (Bmt), L-alpha-aminobutyric acid (Abu), sarcosine and N-methylation at few residues. Peptides with certain other chemical modifications were also screened for its immunosuppressive property (Figure 3C). This modification enhances the stability of peptides. The advantage of using peptide as therapeutic over small molecule is its adequate action by acting on specific molecular target. In most of the cases, over reactive T and B cells have to be suppressed. There are others peptides that act on certain potassium ion channel or specific interleukins as IL-10, IL-2 etc. as well as tumor necrosis factor. Figure 3D shows different targets to achieve immunosuppression by ImmunoSP. Thorough analysis revealed an interesting observation that most of the peptides originated from toxin proteins usually are potassium ion channel blocker such as Vm 24, OSK-1 and their analogues, Margatoxin etc. In general, lymphocyte proliferation inhibition by any pathway is the most common approach to suppress the immune system. Structural information of around 400 unique immunosuppressive peptides is stored in ImmunoSPdb and is also available for the download. Its analysis revealed the existence of diverse secondary structure such as helix, sheet etc. Figure 4 represent one of the example of diversified structures of various immunosuppressive peptide, such as margatoxin has helix as well as β-sheet (Figure 4A), cycloamanide B is an example of cyclic peptide (Figure 4B), a peptide named as SEQ ID30 having sequence RACIDTIPKSRCTAFQCKHSMKYRLSFCRKTCGTC exhibits helical structure (Figure 4C), a peptide derived from myelin basic protein termed as Ac1-9 has been acetylated at N-terminal (Figure 4D) and a peptide named H17, derived from human endogenous retrovirus has amidation at C-terminal end.

Compositional analysis revealed the percentage of amino acid differences with several other functional therapeutic peptides such as anti-cancer, anti-tubercular, cell penetrating etc. (28–30). Beside this, pro- and anti-inflammatory peptides also exhibit compositional differences with immunosuppressive peptides (31, 32). Immunosuppressive peptides have more alanine and leucine as compared to other amino acids, whereas histidine, methionine, asparagine and tryptophan are least favorable. Alanine, cysteine, asparagine, arginine and valine constitute the major compositional difference between immunosuppressive and pro-inflammatory peptides. Similarly percentage composition of alanine, cysteine, glutamate, leucine, asparagine, arginine and valine differentiates between immunosuppressive and anti-inflammatory peptides (Supplementary figure 1).

### Limitation and update of ImmunoSPdb

ImmunoSPdb is a manually curated database i.e. the data have been curated by reading journal articles and patents. In order to preserve the scientific record, provenance of each data has also been enlisted in form of reference i.e. PubMed ID or patent number. We hope the accuracy of data must be at the maximum and of higher quality, but human error cannot be neglected. To minimize the error, we have thoroughly checked the data after curation as well as provided an easy user interface to submit the corrected data. We have tried to compile maximum information, but there are 23 entries, in which detailed amino acid sequence information was not available. We have also stored the predicted structure of immunosuppressive peptide, but some peptides having complicated modifications cannot be predicted due to non-availability of force-field libraries for such modifications.

This database will be updated after adequate time interval; beside this researcher can submit new experimentally validated immunosuppressive peptides and their relevant information. We will confirm its validity to maintain the quality of database.

## Discussion

Despite so many advances and research in immunology, the immunosuppressive regimen remains ambiguous. Although immunosuppression was first introduced in late 1950s and early 1960s by using cytostatic drugs and antimetabolites to control neoplastic cells proliferation, the same decade witnessed many studies, which established azathioprine as an effective immunosuppressive agent in preventing kidney allograft rejections (33–35). In 1980s, calcineurin inhibitors, cyclosporine and tacrolimus were recognized as effective immunosuppressive agents, but their mechanisms were remained opaque for longer time. The basic concept of immunosuppression remains same as interference at various stages of immune response such as destruction of immunocompetent cells, suppression of precursor cells formation, inhibition of purine, pyrimidine and proteins synthesis thus suppressing the proliferation and differentiation of lymphocytes and monocytes (6, 36). But most of the immunosuppressive drugs lack some sort of specificity and hence exhibit life-threatening side effects (8).

In past two decades, peptides have been emerged as promising therapeutic candidates due to its specificity, oral activity, less toxicity and presumed bioavailability. Several databases constituting various therapeutic peptides such as cell-penetrating, anti-tubercular, anti-cancer, tumor-homing, anti-parasitic, anti-hypertensive etc. have been created in the past decade (14, 16, 17, 37–40). These databases provide exhaustive information as well as comprehensive dataset for developing various machine learning models, such as MLCPP, CPPred-FL and CellPPDMod for predicting cell penetrating peptides, MLACP, ACPred-FL and AntiCP for designing of anticancerous peptides, mAHTPred, PAAP and AHTpin for anti-hypertensive peptides, PIP-EL for proinflammatory peptide, AIPpred for anti-inflammatory peptide prediction etc. (28, 31, 32, 41–48). These methods aid the researchers in designing of newer and effective peptide based therapeutic entity.

Beside the traditional approach, now peptide-based immunotherapy and vaccine designing are more fascinating and promising (49–52). Few peptide-based immunosuppressive drugs such as cyclosporine are now in the market. THPdb, a database of Food and Drug Administration (FDA)-approved therapeutic peptides and proteins, enlist 53 entries of 31 unique immunosuppressive peptide or protein based therapeutic entity, which got FDA approval in the past (9). Beside this, many peptides have shown promising immunosuppressive activity and are in advance stage of clinical development for inflammatory diseases (53).

Evolution, natural selection and tendency to fight for survival resulted into plethora of toxins in many living organisms, used for their defense as well as predation. The peptides derived from these toxins are optimized for precise functions by acting on a specific target. So understanding of structure and nature of these naturally occurring peptides as well as their target and mechanism by which they suppress the immune system is unavoidable to hasten new promising immunosuppressive therapy. Therefore, ImmunoSPdb will be very helpful for scientific community aiming to develop promising immunosuppressive therapy.

## Author contributions

S.S.U., P.A., M.S. and P.K.P. manually collected and curated all the data. S.S.U. and P.A. predicted and analyzed the structures. S.S.U. developed the web interface. S.S.U. and G.P.S.R. prepared the manuscript. G.P.S.R. conceived the idea, planned and coordinated the entire project.

## Supplementary Material

Supplementary Data

## Notes

**Database URL**: http://webs.iiitd.edu.in/raghava/immunospdb/
