# Supplementary material for: ImmunoSPdb: an archive of immunosuppressive peptides
Source: Database (Oxford). 2019 Feb 8;2019:baz012. doi: 10.1093/database/baz012 (PMC6367516; doi:10.1093/database/baz012)

**ImmunoSPdb: An Archive of Immunosuppressive Peptides**

**Salman Sadullah Usmani^1,2^, Piyush Agrawal^1,2^, Manika Sehgal^2^, Pradeep Kumar Patel^2^ and Gajendra P.S. Raghava^1,2,^***

^1^ Department of Computational Biology, Indraprastha Institute of Information Technology, Delhi

^2^ Bioinformatics Centre, CSIR-Institute of Microbial Technology, Chandigarh, India

* Corresponding authors

Gajendra P.S. Raghava, Head Department of Computational Biology, Indraprastha Institute of Information Technology, New Delhi 110020, India, Phone: +91-11-2690744

E-mails : [raghava@imtech.res.in](mailto:raghava@imtech.res.in); [raghava@iiitd.ac.in](mailto:raghava@iiitd.ac.in);

Supplementary Figure 1. Comparison of percentage amino acid composition of various functional peptides with respect to immunosuppressive peptide.


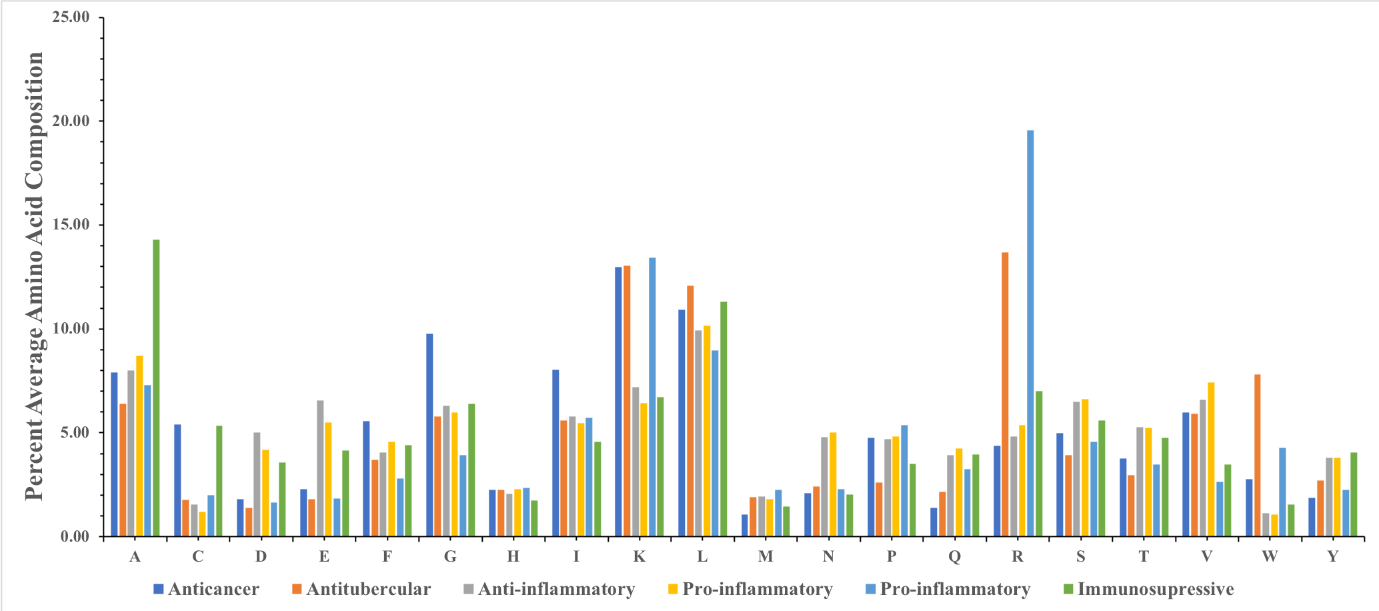

Supplement: Supplementary Data [file supplemantary_baz012_supp.docx]
